# Errata

**Published:** 2012-12

**1.** **Iranian Journal of Microbiology** (2010) December 2 (4):198–209..

The accession number FJ870987 mentioned in the paper entitled **“Molecular epidemiology of zoonotic streptococcosis/lactococcosis in rainbow trout (Oncorhynchus mykiss) aquaculture in Iran”** written by Haghighi Karsidani S, Soltani M, Nikbakhat-Brojeni G, Ghasemi M, and Skall HF (Iran J Microbiol. 2010 December; 2(4): 198–209) is a mistake and so the correct accession number is Ir-D GQ850377.

**2.** **Iranian Journal of Microbiology** (2012) June 4 (2): 70–74.

The accession number FJ870987 mentioned in the paper entitled **“Development of a Reverse Line Blot Hybridization method for Detection of some Streptococcal/Lactococcal Species, the causative agents of Zoonotic Streptococosis/Lactococosis in farmed fish”** written by **Soltani M, Pirali E, Shayan P, Eckert B, Rouholahi S, Sadr Shirazi N** (Iranian Journal of Microbiology (2012) June 4 (2): 70–74.) is a mistake. The correct accession number is Ir-D GQ850377.

